# Real-world effectiveness of IDegLira compared with intensified conventional insulin therapy in adults with type 2 diabetes: a retrospective cohort study

**DOI:** 10.1186/s12902-022-01139-8

**Published:** 2022-09-14

**Authors:** Sándor Szépkúti, Szilvia Bandur, Gábor Kovács, Tamás Ferenci, Márk M. Svébis, Piroska Turbucz, Ádám G. Tabák

**Affiliations:** 1 Diabetology, Pest County Flór Ferenc Hospital, Kistarcsa, Hungary; 2 Physiological Controls Research Center, Óbuda University, Budapest, Hungary; 3 Department of Statistics, Corvinus University of Budapest, Budapest, Hungary; 4 Department of Internal Medicine and Oncology, Semmelweis University Faculty of Medicine, Üllői út 26, Budapest, H1085 Hungary; 5 Department of Public Health, Semmelweis University Faculty of Medicine, Üllői út 26, Budapest, H1085 Hungary; 6 Department of Epidemiology and Public Health, University College London, London, UK

**Keywords:** Insulin therapy; liraglutide; type 2 diabetes mellitus, GLP-1 agonists, Basal insulins, Cohort study

## Abstract

**Background:**

IDegLira is a fixed-ratio combination of insulin degludec and liraglutide with proven efficacy against simpler regimens and non-inferiority against basal-bolus insulin therapy. However, the evaluation of its real-world effectiveness is hindered by technical issues and requires further exploration. Thus we aimed to compare effectiveness of insulin degludec/liraglutide (IDegLira) versus intensified conventional insulin therapy (ICT) for type 2 diabetes in a real-world setting.

**Methods:**

This retrospective cohort study from an outpatient clinic in Hungary included people who initiated IDegLira due to inadequate glycaemic control (HbA1c > 7.0% [53.0 mmol/mol]) with oral and/or injectable antidiabetic drugs. Data were compared with a historical cohort who initiated ICT. Outcomes included HbA1c, body weight, and hypoglycaemia differences over 18 months of follow-up.

**Results:**

Data were included from 227 and 72 people who initiated IDegLira and ICT, respectively. Estimated mean difference (MD) in HbA1c at 18 months favoured IDegLira versus ICT (MD 0.60, 95% CI 0.88–0.32 [MD 6.6 mmol/mol, 95% CI 9.6–3.5]). More people reached target HbA1c ≤7.0% (53.0 mmol/mol) with IDegLira than ICT (odds ratio 3.36, 95% CI 1.52–7.42). IDegLira treatment was associated with weight loss compared with gain for ICT (MD 6.7 kg, 95% CI 5.0–8.5). The hazard ratio for hypoglycaemia comparing IDegLira with ICT was 0.18 (95% CI 0.08–0.49).

**Conclusions:**

Treatment with IDegLira over 18 months resulted in greater HbA1c reductions, weight loss versus gain, and a lower rate of hypoglycaemia versus ICT in people with type 2 diabetes.

**Supplementary Information:**

The online version contains supplementary material available at 10.1186/s12902-022-01139-8.

## Background

Type 2 diabetes is a progressive disease characterized by a continuous decline in beta-cell function and insulin sensitivity starting years before diagnosis [1, 2]. Due to the progressive nature of type 2 diabetes, insulin therapy often becomes necessary after treatment with oral anti-diabetic drugs (OADs) [3].

Long-acting basal insulins improve glycaemic control through decreasing fasting plasma glucose (FPG) and are used alone or in combination with OADs in type 2 diabetes uncontrolled with non-insulin therapies [4, 5]. Although early initiation of basal insulin therapy may confer beta-cell protection [6] and decrease hepatic glucose production, there are barriers to insulin initiation that include fear of hypoglycaemia and weight gain [7].

Glucagon-like peptide-1 receptor agonists (GLP-1 RAs) are incretin mimetics that both stimulate glucose-dependent insulin release and suppress glucagon secretion, leading to reduced blood glucose levels without affecting defence mechanisms to hypoglycaemia. Additionally, GLP-1 RAs slow gastric emptying, increase satiation, and reduce appetite, all of which may lead to body weight loss [8]. The complementary mechanisms of GLP-1 RAs and insulins allow for a combined treatment that provides more effective glycaemic control than either component alone, with less weight gain and a lower risk for hypoglycaemia compared with basal insulins [9, 10].

IDegLira is a fixed-ratio combination of insulin degludec and liraglutide. Its efficacy was studied through the DUAL clinical trial programme [11–18]. In general, IDegLira showed improved efficacy (HbA1c reduction) versus placebo [14], GLP-1 RAs [19], and basal insulins [12, 15], and non-inferiority against basal–bolus therapy [17].

As most DUAL trials compared IDegLira with simpler regimens (e.g. a single injectable antihyperglycaemic medication with/without OADs) and real-world studies often utilize single-arm designs, it is challenging to determine whether people in routine practice are achieving expected outcomes with IDegLira when compared with more complex regimens. The aim of this real-world, retrospective cohort study (with a historical control group) was to compare the effectiveness of IDegLira with that of intensified conventional insulin therapy (ICT) in people with inadequately controlled type 2 diabetes on OADs and/or GLP-1 RAs and/or basal insulin therapy.

## Methods

### Setting and participants

All participants had type 2 diabetes and were followed up at the outpatient clinic of Pest County Flór Ferenc Hospital, Hungary. Participants initiated IDegLira (6/NOV/2015–2/JUL/2018) or ICT (12/FEB/2012–23/AUG/2016) at their physicians’ discretion after failing to achieve HbA1c ≤7.0% (53.0 mmol/mol) after at least 3 months of treatment with OADs and/or GLP-1 RA and/or basal insulin. Data were derived from electronic health records at baseline (treatment initiation) and at approximately 3, 6, 12, and 18 months thereafter. For study inclusion, participants needed data at baseline and ≥ 1 follow-up visit.

For people previously on insulin at doses > 16 IU or on liraglutide, IDegLira was initiated at 16, otherwise at 10 dose steps [20]. Further titration (up to a maximum of 50 dose steps) was done 1–2 times weekly, based on a 3-day average of fasting self-monitored blood glucose (SMBG) with a local target of 5.0–7.0 mmol/L. When IDegLira treatment was initiated, all previously used antidiabetic medications except for metformin were discontinued.

The historical comparison arm included people who initiated ICT (NPH insulin once or twice daily and short-acting human insulin three times daily). Initial dose was based on HbA1c and body weight according to local recommendations [21]. Doses were titrated 1–2 times weekly to achieve fasting and pre-prandial SMBG of 5.0–7.0 mmol/L and a bedtime SMBG of 6.0–7.5 mmol/L. The ICT group continued to receive metformin if this was part of their previous treatment. Other non-insulin antihyperglycaemic medications were discontinued.

### Ethics statement

All study related procedures have been performed in accordance with the ethical standards laid down in the 1964 Declaration of Helsinki and its later amendments. Local ethical approval was obtained from the Ethics Committee of Pest County Flór Ferenc Hospital, Kistarcsa, Hungary; Registration number: 119/17/2017. The need for informed consent was waived in accordance with Hungarian laws by the Ethics Committee of Pest County Flór Ferenc Hospital, Kistarcsa, Hungary.

### Endpoints and co-variables

Key study endpoints included the estimated mean difference (MDs) between the IDegLira and ICT groups in HbA1c, body weight and insulin dose, and the hazard ratio (HR) for incident hypoglycaemia. In addition, a series of target-based composite endpoints were assessed including the achievement of HbA1c ≤7.0% (53.0 mmol/mol) (1) without hypoglycaemia, (2) without weight gain, and (3) with neither hypoglycaemia nor weight gain.

The following data were collected at baseline: sex, age, anthropometric data, diabetes duration, HbA1c, and antihyperglycaemic medications. The following data were recorded at each visit: HbA1c, body weight, insulin dose on the day prior to the visit, incidence of hypoglycaemia since last visit, and antihyperglycaemic medications. HbA1c was measured in the central hospital laboratory, which is accredited by the Hungarian Association for Clinical Chemistry. HbA1c was measured by turbidimetric inhibition immunoassay until 23/06/2015 (COBAS c513, Roche Diagnostics Ltd., Rotkreuz, Switzerland) and using high-performance liquid chromatography (24/06/2015–11/10/2016 using VARIANT II TURBO HbA1c Kit–2.0, Bio-Rad Hungary Ltd., Budapest, Hungary and using Bio-Rad D-100, Bio-Rad Hungary Ltd., Budapest, Hungary) thereafter. Hypoglycaemia was defined as SMBG ≤3.9 mmol/L or with typical symptoms that resolved following carbohydrate consumption. All patients were instructed to record all self-monitored blood glucose values and any event with symptoms suggestive of a hypoglycaemia in their diary.

### Statistical methods

For descriptive analyses, categorical variables are presented as counts and continuous variables are presented as mean ± standard deviation (SD). For the comparison of baseline characteristics, 2-sample t-tests and Chi-square tests were used as appropriate.

For all outcomes except hypoglycaemia, longitudinal data were modelled via generalized least squares with an identity link for continuous outcomes and a logistic link for dichotomous outcomes. The models included gender, age, duration of diabetes, BMI (except for body weight modelling, in which baseline body weight was used) and baseline HbA1c as variables. The two treatment groups were first analysed separately, as baseline parameters could not be matched, followed by multivariate models that compared treatment groups while adjusting for potentially confounding differences. The values used for adjustment in the multivariate model were the modal/median values for the whole sample. The exact date of the visit was used in the multivariate models. The elapsed time to visit and the treatment group and their interaction were included as covariates; this allowed groups to have different effects on endpoints over time. Continuous variables were included with restricted cubic splines to allow for a flexible effect. The correlation structure was chosen from three possible structures by minimizing the Akaike Information Criterion (constant correlation, unstructured correlation matrix, continuous-time first-order autoregressive correlation structure). This accounted for the longitudinal nature, i.e. the intra-individual correlation of measurements over time. MDs between the two groups at given times were calculated using contrasts where *P-*values were not adjusted for multiplicity but confidence intervals (CIs) were simultaneous [22]. Calculations were carried out in R v3.6.1 [23] using package rms v5.1–3.1 (https://cran.r-project.org/web/packages/rms/rms.pdf) and package icenReg v2.0.13 (https://cran.r-project.org/web/packages/icenReg/icenReg.pdf).

Hypoglycaemia was modelled as interval-censored survival data, i.e. the time to first hypoglycaemic event, the interval was from the visit before the first event to the first visit when hypoglycaemia was reported. For those not reporting hypoglycaemia during the study, the interval was recorded as the time of the last visit to infinity. Multivariate modelling was performed using a semi-parametric model for interval-censored data with Cox proportional hazards.

Binary endpoints, i.e. dichotomized HbA1c and composite targets, were modelled by logistic regression with cluster-robust Huber-White (sandwich) covariance estimation to account for intra-individual correlations.

In addition, we performed two sensitivity analyses. First, we further adjusted models for baseline medication use (metformin, sulphonylurea, DPP-4 inhibitor, GLP-1 receptor agonist, and insulin). Second, we restricted the analysis to baseline users of metformin.

## Results

### Baseline characteristics

In total, 227 people who initiated IDegLira (630 outpatient visits, 46 people with 4 visits) and 72 people who initiated ICT (259 outpatient visits, 64 people with 4 visits) were included, with a mean (SD) follow-up of 1.48 (0.21) years. The IDegLira and ICT groups were similar (*P* ≥ 0.05) for the following baseline characteristics: age, body weight, BMI and previous insulin use. However, we found significant between-group differences in other important baseline characteristics: people in the IDegLira group tended to have a longer diabetes duration (MD [SE]: 2.3[0.9] years), lower HbA1c (0.7[0.2]% [7.7[2.2] mmol/mol]) and FPG (3.5[0.6]mmol/L). Furthermore, there were differences in the baseline use of non-insulin antihyperglycaemic medications. All but one person in the IDegLira group were treated with metformin vs. 65.3% of the ICT group. Similarly, fewer people received sulfonylureas in the IDegLira group than in the ICT group, while GLP-1 RAs and dipeptidyl peptidase 4 inhibitors were more frequently used in the IDegLira group (Table 1).

**Table 1:** Baseline characteristics by treatment group

Characteristic	IDegLira group (***n*** = 227)	ICT group (***n*** = 72)	***P*** value
Male, n (%)	122 (53.7)	36 (50.0)	0.59
Age, years	61.2 ± 9.4	61.0 ± 9.5	0.86
Weight, kg	94.9 ± 20.8	90.3 ± 20.8	0.11
Body mass index, kg/m^2^	33.3 ± 5.8	31.9 ± 6.9	0.086
Diabetes duration, years	11.0 ± 6.7	8.6 ± 5.3	0.007
HbA1c, %	8.6 ± 1.2	9.3 ± 1.5	< 0.0001
HbA1c, mmol/mol	70.2 ± 13.3	78.2 ± 16.8	
Fasting plasma glucose, mmol/L	10.6 ± 3.0	14.1 ± 4.7	< 0.0001
Previous antihyperglycaemic medications, n (%)			
Metformin	226 (99.6)	47 (65.3)	< 0.0001
Sulphonylurea	127 (55.9)	59 (81.9)	< 0.0001
DPP-4 inhibitor	117 (51.5)	26 (36.1)	0.03
GLP-1 receptor agonist	23 (10.1)	1 (1.4)	0.013
Insulin	36 (15.9)	10 (13.9)	0.85
Other	5 (2.2)	7 (9.7)	0.01

Data are mean ± standard deviation or n (%). *P* values were based on a two-sample t-test or Chi-square test, as appropriate

% percentage of people in the treatment group, *DPP-4* dipeptidyl peptidase 4, *GLP-1* glucagon-like peptide 1, *ICT* intensified conventional insulin treatment, *IDegLira* insulin degludec/liraglutide, *n* number of people in the treatment group

### Glycaemic control

**Fig. 1:**
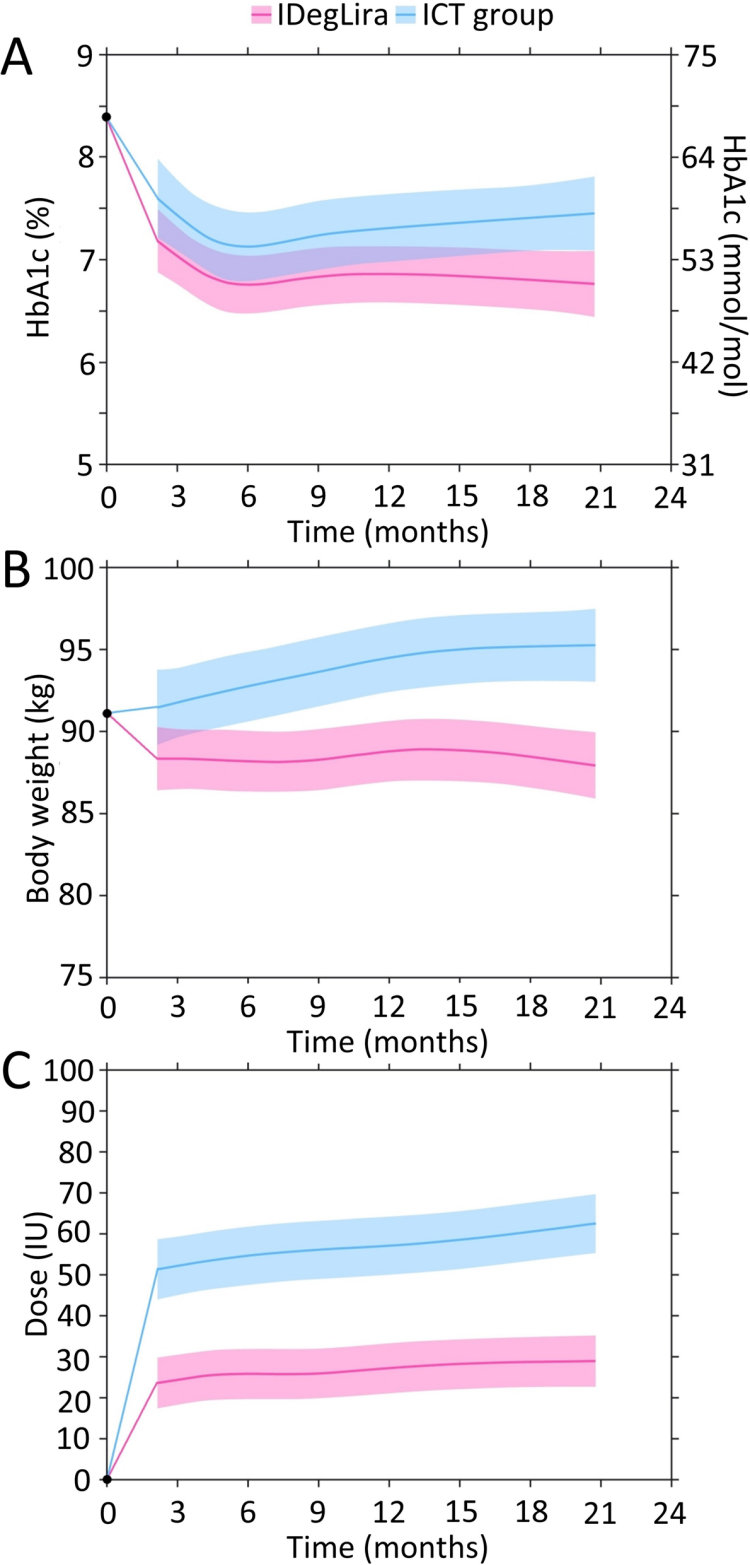
HbA1c (**a**), weight (**b**) and (**c**) insulin dose trajectories with IDegLira and ICT during follow-up. Estimated for a person with the following baseline characteristics: 62 years of age, HbA1c of 8.4% (68.3 mmol/mol), diabetes duration of 9.1 years, body weight of 91 kg, body mass index of 32.0 kg/m^2^ using generalized least squares regression (*n* = 299 people, *n* = 889 observations). The coloured lines represent the treatment estimates, while the shaded areas show 95% confidence intervals around the estimated values. ICT, intensified conventional insulin treatment; IDegLira, insulin degludec/liraglutide; IU, insulin unit

According to the estimated trajectories during follow-up, HbA1c decreased from baseline and reached its nadir at approximately 6 months for both treatments (Fig. 1a). While there seemed to be an increase in HbA1c afterwards in the ICT group, HbA1c remained more stable in the IDegLira group, which is reflected in the increasing MDs between groups after 6 months of follow-up (6 months 0.37% [4.0 mmol/mol]; 12 months 0.45% [4.9 mmol/mol]; 18 months 0.60% [6.6 mmol/mol]) (Table 2). Overall, the differences in HbA1c between groups were significant at all timepoints, in favour of IDegLira after differences in baseline characteristics were taken into account (*P* < 0.01 for all).

**Table 2:** Estimated mean differences in HbA1c, weight, and daily insulin between IDegLira and ICT during follow-up

	3 months			6 months			12 months			18 months		
	MD	95% CI	***P*** value	MD	95% CI	***P*** value	MD	95% CI	***P*** value	MD	95% CI	***P*** value
HbA1c (%)	0.40	0.10; 0.70	0.0014	0.37	0.07; 0.67	0.0024	0.45	0.17; 0.72	0.0001	0.60	0.32; 0.88	< 0.0001
HbA1c (mmol/mol)	4.4	1.1; 7.7		4.0	0.8; 7.3		4.9	1.9; 7.9		6.6	3.5; 9.6	
Body weight (kg)	3.4	1.7; 5.2	< 0.0001	4.6	2.8; 6.3	< 0.0001	5.7	4.0; 7.4	< 0.0001	6.7	5.0; 8.5	< 0.0001
Insulin dose (IU)	27.8	22.6; 33.0	< 0.0001	28.9	23.7; 34.0	< 0.0001	29.9	24.8; 35.0	< 0.0001	31.8	26.6; 37.0	< 0.0001

Estimated for a person with the following baseline characteristics: 62 years of age, HbA1c of 8.4% (68.3 mmol/mol), diabetes duration of 9.1 years, body weight of 91 kg, body mass index of 32.0 kg/m^2^ using generalized least squares regression (*n* = 299 people, *n* = 889 observations). MD [95% CI] presented for IDegLira – ICT

*CI* confidence interval, *ICT* intensified conventional insulin treatment, *IDegLira* insulin degludec/liraglutide, *IU* insulin units, *MD* mean difference

**Fig. 2:**
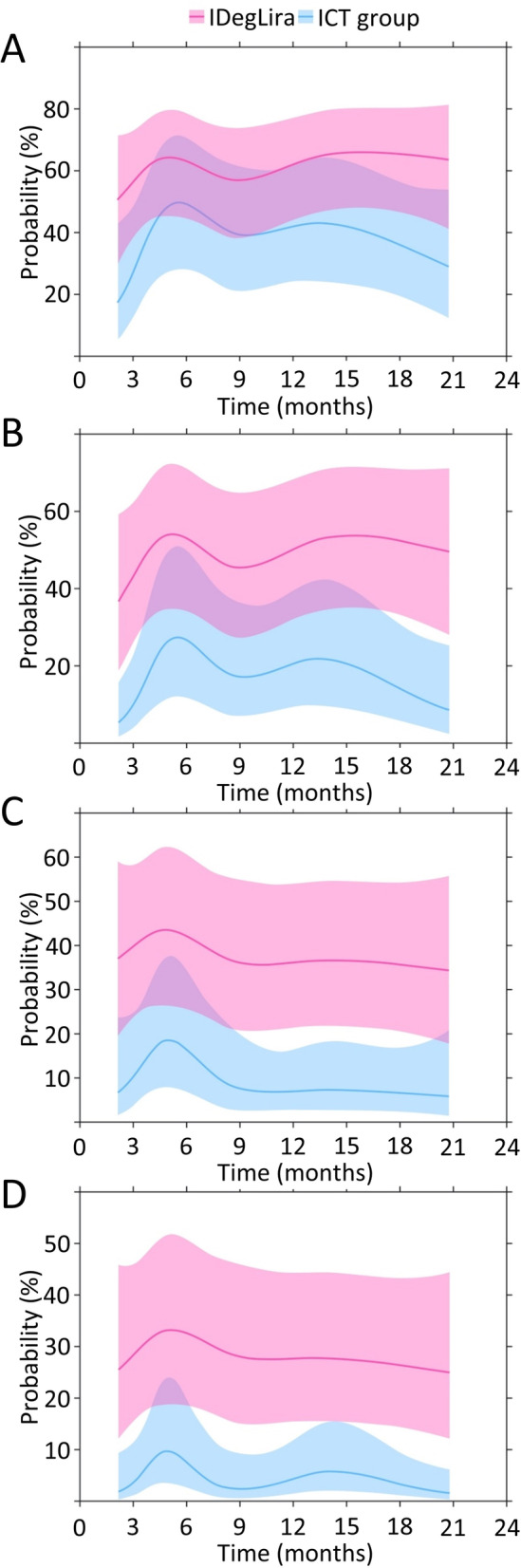
Estimated probabilities of achieving categorical outcomes with IDeglira and ICT during follow-up. **a** – HbA1c ≤7.0% (53.0 mmol/mol). **b** – HbA1c ≤7.0% (53.0 mmol/mol) without hypoglycaemia. **c** – HbA1c ≤7.0% (53.0 mmol/mol) without weight gain. **d** – HbA1c ≤7.0% (53.0 mmol/mol) without hypoglycaemia or weight gain. Estimated for a person with the following baseline characteristics: 62 years of age, HbA1c of 8.4% (68.3 mmol/mol), diabetes duration of 9.1 years, body weight of 91 kg, body mass index of 32.0 kg/m^2^ using generalized least squares regression (*n* = 299 people, *n* = 889 observations). The coloured lines represent the treatment estimates, while the shaded areas show 95% confidence intervals around the estimated values. ICT, intensified conventional insulin treatment; IDegLira, insulin degludec/liraglutide

According to models adjusted for baseline differences, over half of the IDegLira group and substantially fewer in the ICT group reached target HbA1c ≤7.0% (53.0 mmol/mol) during follow-up (Fig. 2a). While there was no significant difference in the odds of reaching the HbA1c target at 6 months, people in the IDegLira group had a 2–3 times greater odds of reaching this target at the other timepoints (Table 3).

**Table 3:** Estimated odds ratios for achievement of categorical outcomes with IDegLira group versus ICT during follow-up

Categorical outcome	3 months			6 months			12 months			18 months		
	OR	95% CI	***P*** value	OR	95% CI	***P*** value	OR	95% CI	***P*** value	OR	95% CI	***P*** value
HbA1c ≤7.0% (53.0 mmol/mol)	3.52	1.36; 9.09	0.0011	1.77	0.67; 4.64	0.145	2.26	1.05; 4.88	0.0086	3.36	1.52; 7.42	0.00015
HbA1c ≤7.0% (53.0 mmol/mol) without hypoglycaemia	6.83	2.59; 18.00	< 0.0001	3.10	1.18; 8.11	0.0039	3.90	1.70; 8.93	< 0.0001	6.71	2.75; 16.37	< 0.0001
HbA1c ≤7.0% (53.0 mmol/mol) without weight gain	5.77	1.97; 16.89	< 0.0001	3.67	13.7; 9.80	0.0011	7.48	2.80; 19.94	< 0.0001	7.81	2.73; 22.31	< 0.0001
HbA1c ≤7.0% (53.0 mmol/mol) without hypoglycaemia or weight gain	10.46	2.76; 39.59	< 0.0001	5.98	1.91; 18.66	0.0001	8.55	2.86; 25.5	< 0.0001	10.47	3.27; 35.50	< 0.0001

Estimated for a person with the following baseline characteristics: 62 years of age, HbA1c of 8.4% (68.3 mmol/mol), diabetes duration of 9.1 years, body weight of 91 kg, body mass index of 32.0 kg/m^2^ using generalized least squares regression (n = 299 people, n = 889 observations). OR [95% CI] presented for IDegLira/ICT

*CI* confidence interval, *ICT* intensified conventional insulin treatment, *IDegLira* insulin degludec/liraglutide, *OR* odds ratio

### Body weight

Body weight decreased slightly from baseline to 3 months in the IDegLira group and was relatively stable thereafter until 18 months of follow-up (Fig. 1b). For people in the ICT group, body weight increased gradually from baseline to month 18, resulting in the group trajectories diverging over time. The estimated MD (adjusted for baseline differences) between groups was significant at all timepoints, reaching 6.7 kg (95% CI 5.0–8.5) at 18 months of follow-up, in favour of IDegLira (*P* < 0.0001) (Table 2).

### Insulin dose

The daily insulin dose with IDegLira was significantly lower than with ICT at each timepoint, with the discrepancy increasing over time to reach an MD of 31.8 IU (95% CI 26.6–37.0) at month 18 in models adjusted for baseline differences (Fig. 1c; Table 2).

### Incident hypoglycaemia

During follow-up, there were 14 episodes of hypoglycaemia in the IDegLira group and 24 in the ICT group. The adjusted HR (IDegLira/ICT) for hypoglycaemia was 0.18 (95% CI 0.08–0.49), indicating a lower incidence of hypoglycaemia with IDegLira than with ICT.

### Composite outcomes

During follow-up, all three composite outcomes were reached more frequently by people in the IDegLira group than by those in the ICT group (Fig. 2 b-d). People had 3–8 times greater odds of reaching the target HbA1c ≤7.0% (53.0 mmol/mol) without either hypoglycaemia or weight gain in the IDegLira group than in the ICT group, with the largest point estimates at 18 months of follow-up: adjusted odds ratios (ORs) of 6.7 (95% CI 2.8–16.4) and 7.8 (95% CI 2.7–22.3), respectively (Table 3). The odds of achieving the 3-point composite outcome were higher with IDegLira: people had 6–10 times greater odds of reaching the target HbA1c ≤7.0% (53.0 mmol/mol) without hypoglycaemia and without weight gain in the IDegLira group than in the ICT group after adjustment for baseline differences.

### Sensitivity analyses

Both sensitivity analyses (the one adjusted for baseline antidiabetic medications, the one restricted to baseline metformin users) confirmed the results of the main analysis with similar effect sizes and somewhat wider confidence intervals (Table S1-S4, Additional File 1).

## Discussion

In this real-world study over 18 months of follow-up, treatment with IDegLira resulted in a greater reduction in HbA1c (MD 0.6% [6.6 mmol/mol] lower), a relative reduction in body weight (MD 6.7 kg lower), a lower insulin dose (MD 31.8 IU/day lower), and an 82% lower risk of hypoglycaemia when compared with ICT. HbA1c decreased from baseline in both treatment groups, reaching a nadir with mean estimated values of 6.8% (50.9 mmol/mol) with IDegLira and 7.1% (54.1 mmol/mol) with ICT at 6 months.

These HbA1c improvements are in accordance with those observed over 26 weeks in the DUAL trials: 1.4–1.9% (15.3–20.8 mmol/mol) with IDegLira, and 1.5% (16.4 mmol/mol) with basal–bolus therapy [11–15, 17, 18]. Our finding that the decrease in HbA1c was larger with IDegLira than with ICT contrasts with the DUAL VII findings, where reductions were similar [17]. This contrast could most likely be explained by the different patient characteristics between our study and the DUAL VII trial. While only ~ 15% of our participants were insulin users at baseline, all DUAL VII participants were on basal insulin treatment at baseline suggesting a more advanced disease status where GLP-1 receptor agonists may be less effective. It is also possible that the fact that titration targets were the same for the ICT and IDegLira groups in our study, while they were more relaxed in the ICT group in DUAL VII, explained some of the observed differences. It is less likely that the more relaxed titration targets in our study or the use of human insulins rather than insulin analogues would explain these differences. In DUAL VII, 66% of people achieved HbA1c < 7.0% (53.0 mmol/mol) with IDegLira, in the other DUAL trials this ranged 56 to 81%, consistent with our finding of over 60% [11–15, 17, 18].

IDegLira treatment was associated with an approximate 3 kg reduction in body weight from baseline that was apparent from 3 months’ follow-up and persisted throughout the study. In contrast, people in the ICT group gained weight over follow-up, leading to increasing MDs in body weight, rising from 3.4 kg to 6.7 kg. Weight loss with IDegLira is somewhat larger than that observed in the DUAL trials (− 2.8 kg to + 2.0 kg) [11–15, 17, 18].. The greater reduction in body weight observed with IDegLira versus ICT in our study compared with the DUAL trials may be due to the lower daily doses of IDegLira used in our study (22.2 IU at 6 months compared with 41 IU and 40 IU at 26 weeks in DUAL V and DUAL VII, respectively). The weight gain observed with ICT treatment (~ 2 kg) was similar to that observed with basal–bolus insulin in DUAL VII (2.6 kg) or with basal insulins in DUAL I, II, V, and VIII (0–3 kg) [12, 15, 18, 19].

There was a substantially lower risk (HR 0.18, 95% CI 0.08–0.49) of incident hypoglycaemia with IDegLira versus ICT in our real-world study, which was somewhat lower than the estimated risk ratio of 0.39 (95% CI 0.29–0.51) for IDegLira versus basal–bolus insulin reported in DUAL VII [17]. Furthermore, the absolute risks are much lower in our study (6.2% for IDegLira vs. 33.3% for ICT) compared to DUAL VII (19.8% vs. 52.6%, respectively) that highlights the differences in baseline characteristics between participants and suggests that direct comparisons cannot be made between these studies. There are also other important design differences between the studies that could bias this comparison and could have effects with unknown directions: (1) a less stringent SMBG target (5.0–7.0 mmol/L) in our study (cf. 4.0–5.0 mmol/L in DUAL VII) [17], (2) similar treatment targets for both arms in our study (cf. less stringent for ICT in DUAL VII), (3) less insulin use in our study at baseline (~ 15% vs. 100% in DUAL VII), (4) a tendency towards lower insulin doses in our real-world study, and (5) different hypoglycaemia definitions that included a higher SMBG threshold (≤3.9 mmol/L) in our study (cf. < 3.1 mmol/L in DUAL VII) [17].

In terms of insulin dose, we found a significantly lower dose (by 28–32 IU/day) at each timepoint in the IDegLira group in comparison with the ICT group. The absolute insulin dose was relatively low in both the IDegLira (~ 30 IU/day) and the ICT (~ 55 IU/day) groups when compared with the end-of-trial dose in DUAL VII (40 and 84 IU/day for IDegLira and basal–bolus insulin, respectively) well reflecting the fact that only ~ 15% our participants used insulin at baseline compared 100% in DUAL VII [17]. When compared more widely with other DUAL trials, the IDegLira doses found in our real-world study (30–45 IU/day) were lower than the end-of-trial doses [11–18]. These differences are most likely due to the more stringent SMBG targets in the DUAL trials [11–18], whereas the doses we observed are likely to better reflect routine practice.

Our findings for the composite outcomes are of particular interest due to the opportunity to investigate the time-course of changes from the repeated data collection. The results suggest that differences in the attainment of composite outcomes between treatment groups (in favour of IDegLira) increase in magnitude over time. At six months, ORs for the attainment of composite outcomes with IDegLira versus ICT in our real-world study were in general agreement with those reported in DUAL VII versus basal–bolus insulin [17]. In addition to supporting the DUAL VII trial findings in routine practice, our findings extend the results of the 32-week DUAL VII trial, providing an insight into trajectories over 18 months of follow-up. In fact, the DUAL VII results for composite outcomes may underestimate the longer-term benefits of IDegLira versus ICT in this population in routine clinical practice.

Our study compared two potential ways of treatment intensification of inadequately controlled type 2 diabetes, however clinicians face other options that should be mentioned. First, there are 2 fixed ratio combinations of basal insulins and GLP-1 receptor agonists using different components of both medications (insulin degludec/liraglutide and insulin glargine/lixisenatide [iGlarLixi]). While there is no direct comparison of these medications, results based on indirect comparisons show equivocal results with some suggesting superiority of IDegLira over iGlarLixi, some suggesting similar efficacy on metabolic control and weight change [24–26]. Given the fact that the use of iGlarLixi was much less frequent at the time of data collection, we limited our analysis to IDegLira, thus potentially limiting heterogeneity in our results. Another option of intensification is initiating a free combination of GLP-1 receptor agonists and basal insulins. This approach was investigated in a meta-analysis of randomized trials showing similar results on all investigated outcomes [27]. In contrast, a large real world analysis suggested similar effect on HbA1c but a more limited effect on weight with the use of fixed ratio combinations. These could be related to the fact that the dose of GLP-1 receptor agonists could be suboptimal for weight change in people on relatively small doses of the fixed ratio combinations [28].

One limitation, common to most real-world studies, is the lack of an active comparator arm, with most utilising a pre/post study design, whereby a placebo effect and the medication effect cannot be definitively separated. Our real-world study included a historical comparison group and our findings are in alignment with previous IDegLira real-world evidence – that initiating IDegLira improved glycaemic control in the magnitude of 0.3–1.7% [29–31] without weight gain (reductions of 0.4–3.1 kg [29–32]) when switching from a variety of previous regimens. While the improvement in glycaemic control tends to be slightly smaller (by 0.3–0.7% [30, 32]) when switching to IDegLira from multiple daily insulin injections, the weight loss tends to be larger (2.4–3.1 kg [30, 31]). A further limitation is selection bias related to the retrospective design of the study. However, this bias is likely to have affected the IDegLira and ICT groups similarly. Another potential limitation is the between-group differences in some baseline characteristics, which may reflect an indication bias: some people may not have been intensified to ICT treatment, while others (e.g. those with HbA1c > 10% [85.8 mmol/mol] or symptomatic hyperglycaemia) received ICT treatment [4]. Indeed, the observed differences in baseline characteristics between the groups (diabetes duration, HbA1c, fasting glucose, body mass index, different use of medications) may point to more severe insulin secretory defect in the ICT group compared to the IDegLira group. While we adjusted for several potential confounders, the possibility of other uncontrolled or unmeasured confounding factors cannot be excluded. One important potential unmeasured confounder is the presence or absence of long term diabetes complications and medications used for their treatment or prevention. Although we had no information on these parameters, in sensitivity analyses, we adjusted for the use of different antidiabetic medications and ran another analysis restricted to only metformin users at baseline. The choice of older (human) insulins as the comparator may be viewed as a limitation. While there were no clinically significant differences in glycaemic control in meta-analyses that compared NPH and short-acting human insulins with long- and short-acting insulin analogues, respectively [33, 34], there was a lower risk of hypoglycaemia with long-acting insulin analogues than with NPH insulin [34]. Hence, there may have been better tolerability and quality of life in a comparator arm of people initiating longer-acting basal and short-acting insulin analogues.

A key strength of real-world studies is the ability to observe the effects of a medication in a broad range of people and under the conditions of routine clinical practice. A strength of our study is the use of a historical control group at a similar disease stage that had initiated a new treatment, thus minimizing any placebo effects. Although our control group was somewhat different from the IDegLira group, we used statistical methodology to control for differences in baseline characteristics. The fact that our main and sensitivity analyses point to the same direction with similar effect sizes supports the robustness of our findings. Our follow-up time (up to 18 months) was greater than that of most randomized controlled trials, (generally 26 weeks) or real-world studies (often 6–12 months), enabling an assessment of longer-term effectiveness. Given the standardized data collection and treatment algorithms leading to similar frequency of dose titration at our centre, the risk of several types of bias frequently found in real world studies could have only a limited effect on our results including bias in classification of or deviation from intervention, bias due to missing data or measurement of outcomes [35].

## Conclusions

To conclude, in this real-world study, treatment with IDegLira resulted in greater reductions in HbA1c, weight loss as compared with weight gain, and a lower rate of hypoglycaemia than with ICT. Although we used less stringent SMBG titration targets that reflected routine practice (cf. the DUAL trials), the overall effectiveness of IDegLira was similar or improved, with 10 times greater odds of achieving target HbA1c without hypoglycaemia and without weight gain with IDegLira versus ICT after 18 months of follow-up.

## Supplementary Information

**Additional file 1: Tables S1-S4.** Results of sensitivity analyses (supplementary tables).

## Data Availability

The datasets generated and/or analysed during the current study are not publicly available due to ethical reasons concerning the lack of the participants’ informed consents but are available from the corresponding author on reasonable request.

## Abbreviations

BMI: Body mass index
CI: Confidence interval
FPG: Fasting plasma glucose
GLP-1 RA: Glucagon-like peptide-1 receptor agonist
HR: Hazard ratio
HbA1c: Glycated haemoglobin
ICT: Intensified conventional insulin therapy
IDegLira: fixed-ratio combination of insulin degludec and liraglutide
iGlarLixi: fixed-ratio combination of insulin glargine and lixisenatide
IU: International unit
MD: Mean difference
NPH: Natural protamine Hagedorn
OAD: Oral anti-diabetic drug
OR: Odds ratio
SD: Standard deviation
SE: Standard error
SMBG: Self-monitored blood glucose

## References

[1] Fonseca VA (2009). Defining and characterizing the progression of type 2 diabetes. Diabetes Care.

[2] Tabak AG, Herder C, Rathmann W, Brunner EJ, Kivimaki M (2012). Prediabetes: a high-risk state for diabetes development. Lancet..

[3] Turner RC, Cull CA, Frighi V, Holman RR (1999). Glycemic control with diet, sulfonylurea, metformin, or insulin in patients with type 2 diabetes mellitus: progressive requirement for multiple therapies (UKPDS 49). UK prospective diabetes study (UKPDS) group. JAMA..

[4] Davies MJ, D'Alessio DA, Fradkin J, Kernan WN, Mathieu C, Mingrone G (2018). Management of Hyperglycemia in type 2 diabetes, 2018. A consensus report by the American Diabetes Association (ADA) and the European Association for the Study of diabetes (EASD). Diabetes Care.

[5] American DA (2021). 9. Pharmacologic approaches to glycemic treatment: standards of medical Care in Diabetes-2021. Diabetes Care.

[6] Weng J, Li Y, Xu W, Shi L, Zhang Q, Zhu D (2008). Effect of intensive insulin therapy on beta-cell function and glycaemic control in patients with newly diagnosed type 2 diabetes: a multicentre randomised parallel-group trial. Lancet..

[7] Khunti S, Khunti K, Seidu S (2019). Therapeutic inertia in type 2 diabetes: prevalence, causes, consequences and methods to overcome inertia. Ther Adv Endocrinol Metab.

[8] Drucker DJ, Nauck MA (2006). The incretin system: glucagon-like peptide-1 receptor agonists and dipeptidyl peptidase-4 inhibitors in type 2 diabetes. Lancet..

[9] Nuffer W, Guesnier A, Trujillo JM (2018). A review of the new GLP-1 receptor agonist/basal insulin fixed-ratio combination products. Ther Adv Endocrinol Metab.

[10] Anderson SL, Trujillo JM (2016). Basal insulin use with GLP-1 receptor agonists. Diabetes Spectr.

[11] Gough SC, Bode BW, Woo VC, Rodbard HW, Linjawi S, Zacho M (2015). One-year efficacy and safety of a fixed combination of insulin degludec and liraglutide in patients with type 2 diabetes: results of a 26-week extension to a 26-week main trial. Diabetes Obes Metab.

[12] Buse JB, Vilsboll T, Thurman J, Blevins TC, Langbakke IH, Bottcher SG (2014). Contribution of liraglutide in the fixed-ratio combination of insulin degludec and liraglutide (IDegLira). Diabetes Care.

[13] Linjawi S, Bode BW, Chaykin LB, Courreges JP, Handelsman Y, Lehmann LM (2017). The efficacy of IDegLira (insulin Degludec/Liraglutide combination) in adults with type 2 diabetes inadequately controlled with a GLP-1 receptor agonist and Oral therapy: DUAL III randomized clinical trial. Diabetes Ther.

[14] Rodbard HW, Bode BW, Harris SB, Rose L, Lehmann L, Jarlov H (2017). Safety and efficacy of insulin degludec/liraglutide (IDegLira) added to sulphonylurea alone or to sulphonylurea and metformin in insulin-naive people with type 2 diabetes: the DUAL IV trial. Diabet Med.

[15] Lingvay I, Perez Manghi F, Garcia-Hernandez P, Norwood P, Lehmann L, Tarp-Johansen MJ (2016). Effect of insulin glargine up-titration vs insulin Degludec/Liraglutide on glycated hemoglobin levels in patients with uncontrolled type 2 diabetes: the DUAL V randomized clinical trial. JAMA..

[16] Harris SB, Kocsis G, Prager R, Ridge T, Chandarana K, Halladin N (2017). Safety and efficacy of IDegLira titrated once weekly versus twice weekly in patients with type 2 diabetes uncontrolled on oral antidiabetic drugs: DUAL VI randomized clinical trial. Diabetes Obes Metab.

[17] Billings LK, Doshi A, Gouet D, Oviedo A, Rodbard HW, Tentolouris N (2018). Efficacy and safety of IDegLira versus basal-bolus insulin therapy in patients with type 2 diabetes uncontrolled on metformin and basal insulin: the DUAL VII randomized clinical trial. Diabetes Care.

[18] Aroda VR, Gonzalez-Galvez G, Gron R, Halladin N, Haluzik M, Jermendy G (2019). Durability of insulin degludec plus liraglutide versus insulin glargine U100 as initial injectable therapy in type 2 diabetes (DUAL VIII): a multicentre, open-label, phase 3b, randomised controlled trial. Lancet Diabetes Endocrinol.

[19] Gough SC, Bode B, Woo V, Rodbard HW, Linjawi S, Poulsen P (2014). Efficacy and safety of a fixed-ratio combination of insulin degludec and liraglutide (IDegLira) compared with its components given alone: results of a phase 3, open-label, randomised, 26-week, treat-to-target trial in insulin-naive patients with type 2 diabetes. Lancet Diabetes Endocrinol.

[20] Novo Nordisk A/S. Xultophy Summary of Product Characteristics 2020 [Available from: https://www.medicines.org.uk/emc/product/3469.

[21] Ge J (2017). Clinical practice guideline – diagnosis, antihyperglycaemic treatment and care of patients with diabetes in adulthood. Diabetologia Hungarica.

[22] Harrell F (2015). Regression modeling strategies: with applications to linear models, logistic and ordinal regression, and survival analysis.

[23] R Core Team. R: A language and environment for statistical computing. R Foundation for Statistical Computing, Vienna 2019 [Available from: http://www.R-project.org/.

[24] McCrimmon RJ, Lamotte M, Ramos M, Alsaleh AJO, Souhami E, Lew E (2021). Cost-effectiveness of iGlarLixi versus iDegLira in type 2 diabetes mellitus inadequately controlled by GLP-1 receptor agonists and Oral Antihyperglycemic therapy. Diabetes Ther.

[25] Evans M, Billings LK, Hakan-Bloch J, Slothuus U, Abrahamsen TJ, Andersen A (2018). An indirect treatment comparison of the efficacy of insulin degludec/liraglutide (IDegLira) and insulin glargine/lixisenatide (iGlarLixi) in patients with type 2 diabetes uncontrolled on basal insulin. J Med Econ.

[26] Cai X, Gao X, Yang W, Ji L (2017). Comparison between insulin degludec/liraglutide treatment and insulin glargine/lixisenatide treatment in type 2 diabetes: a systematic review and meta-analysis. Expert Opin Pharmacother.

[27] Maiorino MI, Chiodini P, Bellastella G, Scappaticcio L, Longo M, Esposito K (2018). Free and fixed-ratio combinations of basal insulin and GLP-1 receptor agonists versus basal insulin intensification in type 2 diabetes: a systematic review and meta-analysis of randomized controlled trials. Diabetes Obes Metab.

[28] Morieri ML, Rigato M, Frison V, Simioni N, D'Ambrosio M, Tadiotto F (2019). Fixed versus flexible combination of GLP-1 receptor agonists with basal insulin in type 2 diabetes: a retrospective multicentre comparative effectiveness study. Diabetes Obes Metab.

[29] Sofra D (2017). Glycemic control in a real-life setting in patients with type 2 diabetes treated with IDegLira at a single Swiss center. Diabetes Ther.

[30] Price H, Bluher M, Prager R, Phan TM, Thorsted BL, Schultes B (2018). Use and effectiveness of a fixed-ratio combination of insulin degludec/liraglutide (IDegLira) in a real-world population with type 2 diabetes: results from a European, multicentre, retrospective chart review study. Diabetes Obes Metab.

[31] Taybani Z, Botyik B, Katko M, Gyimesi A, Varkonyi T (2019). Simplifying complex insulin regimens while preserving good glycemic control in type 2 diabetes. Diabetes Ther.

[32] Melzer-Cohen C, Chodick G, Naftelberg S, Shehadeh N, Karasik A (2020). Metabolic control and adherence to therapy in type 2 diabetes mellitus patients using IDegLira in a real-world setting. Diabetes Ther.

[33] Fullerton B, Siebenhofer A, Jeitler K, Horvath K, Semlitsch T, Berghold A (2018). Short-acting insulin analogues versus regular human insulin for adult, non-pregnant persons with type 2 diabetes mellitus. Cochrane Database Syst Rev.

[34] Monami M, Marchionni N, Mannucci E (2008). Long-acting insulin analogues versus NPH human insulin in type 2 diabetes: a meta-analysis. Diabetes Res Clin Pract.

[35] Morieri ML, Avogaro A, Fadini GP (2020). Long-acting injectable GLP-1 receptor agonists for the treatment of adults with type 2 diabetes: perspectives from clinical practice. Diabetes Metab Syndr Obes.

