# Supplementary material for: Real-world effectiveness of IDegLira compared with intensified conventional insulin therapy in adults with type 2 diabetes: a retrospective cohort study
Source: BMC Endocr Disord. 2022 Sep 14;22:229. doi: 10.1186/s12902-022-01139-8 (PMC9476268; doi:10.1186/s12902-022-01139-8)
Supplement: Supplementary file 1 — Additional file 1: Tables S1-S4. Results of sensitivity analyses (supplementary tables). [file 12902_2022_1139_MOESM1_ESM.pdf]

Real-world effectiveness of IDegLira compared with intensified conventional insulin therapy in adults with type 2 diabetes: a retrospective cohort study

Sándor Szépkúti[1], Szilvia Bandur[1], Gábor Kovács[1], Tamás Ferenci[2,3], Márk M. Svébis[4]; Piroska Turbucz[1], Ádám G. Tabák[4,5,6]

[1] Diabetology, Pest County Flór Ferenc Hospital, Kistarcsa, Hungary

[2] Physiological Controls Research Center, Óbuda University, Budapest, Hungary

[3] Department of Statistics, Corvinus University of Budapest, Budapest, Hungary

[4] Department of Internal Medicine and Oncology, Semmelweis University Faculty of Medicine, Budapest, Hungary

[5] Department of Public Health, Semmelweis University Faculty of Medicine, Budapest, Hungary

[6] Department of Epidemiology and Public Health, University College London, London, United Kingdom

Table S1 Estimated mean differences in HbA1c, weight, and daily insulin between IDegLira and ICT during follow-up

	3 months			6 months			12 months			18 months		
	**MD**	**95% CI**	***P* value**	**MD**	**95% CI**	***P* value**	**MD**	**95% CI**	***P* value**	**MD**	**95% CI**	***P* value**
HbA1c (%)	0.26	-0.07-0.58	0.06	0.24	-0.07-0.56	0.07	0.33	0.03-0.63	0.009	0.48	0.18-0.79	0.0001
HbA1c (mmol/mol)	2.8	-0.7-6.4		2.7	-0.8-6.1		3.6	0.3-6.8		5.3	2-8.6	
Body weight (kg)	3.18	-0.16-6.52	0.03	4.26	0.94-7.58	0.004	5.33	2.04-8.61	0.0003	6.19	2.87-9.5	<0.0001
Insulin dose (IU)	26.3	20.5-32.2	<0.0001	27.4	21.6-33.2	<0.0001	28.5	22.7-34.3	<0.0001	30.4	24.6-36.3	<0.0001

Models adjusted for sex, age, baseline HbA1c, diabetes duration, body weight, body mass index, metformin, sulphonylurea, DPP-4 inhibitor, GLP-1 receptor agonist, and insulin use with generalized least squares regression (n = 299 people, n = 889 observations). MD [95% CI] presented for IDegLira – ICT.

CI, confidence interval; ICT, intensified conventional insulin treatment; IDegLira, insulin degludec/liraglutide; IU, insulin units; MD, mean difference.

Table S2 Estimated odds ratios for achievement of categorical outcomes with IDegLira group versus ICT during follow-up

Categorical outcome	3 months			6 months			12 months			18 months		
	OR	95% CI	*P* value	OR	95% CI	*P* value	OR	95% CI	*P* value	OR	95% CI	*P* value
HbA1c ≤7.0% (53.0 mmol/mol)	3.22	1.11-9.27	0.007	1.72	0.61-4.82	0.20	2.25	0.94-5.36	0.02	3.35	1.41-7.91	0.0005
HbA1c ≤7.0% (53.0 mmol/mol)without hypoglycaemia	5.67	1.84-17.38	0.0002	2.66	0.9-7.85	0.03	3.40	1.33-8.66	0.002	5.85	2.16-15.79	<0.0001
HbA1c ≤7.0% (53.0 mmol/mol) without weight gain	8.03	2.28-28.26	0.0001	4.90	1.61-14.86	0.0005	10.67	3.39-33.48	<0.0001	10.94	3.62-33.02	<0.0001
HbA1c ≤7.0% (53.0 mmol/mol) without hypoglycaemia or weight gain	10.76	2.26-51.01	0.0002	6.21	1.79-21.45	0.0003	8.91	2.81-28.23	<0.0001	10.96	3.11-38.52	<0.0001

Models adjusted for sex, age, baseline HbA1c, diabetes duration, body weight, body mass index, metformin, sulphonylurea, DPP-4 inhibitor, GLP-1 receptor agonist, and insulin use with generalized least squares regression (n = 299 people, n = 889 observations). OR [95% CI] presented for IDegLira/ICT.

CI, confidence interval; ICT, intensified conventional insulin treatment; IDegLira, insulin degludec/liraglutide; OR, odds ratio.

Table S3 Estimated mean differences in HbA1c, weight, and daily insulin between IDegLira and ICT during follow-up

	3 months			6 months			12 months			18 months		
	**MD**	**95% CI**	***P* value**	**MD**	**95% CI**	***P* value**	**MD**	**95% CI**	***P* value**	**MD**	**95% CI**	***P* value**
HbA1c (%)	0.32	-0.05-0.69	0.03	0.32	-0.03-0.66	0.02	0.35	0.03-0.66	0.007	0.52	0.2-0.85	0.0001
HbA1c (mmol/mol)	3.5	-0.6-7.6		3.5	-0.3-7.3		3.8	0.3-7.3		5.7	2.2-9.3	
Body weight (kg)	2.57	-0.98-6.13	0.10	4.10	0.6-7.6	0.008	5.23	1.79-8.67	0.0006	6.28	2.81-9.75	<0.0001
Insulin dose (IU)	27.4	21.2-33.6	<0.0001	29.8	23.7-35.9	<0.0001	30.2	24.1-36.2	<0.0001	32.7	26.6-38.8	<0.0001

Models restricted to baseline metformin users and adjusted for sex, age, baseline HbA1c, diabetes duration, body weight, and body mass index using generalized least squares regression (n = 273 people). MD [95% CI] presented for IDegLira – ICT. MD [95% CI] presented for IDegLira – ICT.

CI, confidence interval; ICT, intensified conventional insulin treatment; IDegLira, insulin degludec/liraglutide; IU, insulin units; MD, mean difference.

Table S4 Estimated odds ratios for achievement of categorical outcomes with IDegLira group versus ICT during follow-up

Categorical outcome	3 months			6 months			12 months			18 months		
	OR	95% CI	*P* value	OR	95% CI	*P* value	OR	95% CI	*P* value	OR	95% CI	*P* value
HbA1c ≤7.0% (53.0 mmol/mol)	5.39	1.83-15.88	0.0001	1.57	0.5-4.85	0.33	2.14	0.86-5.3	0.04	3.08	1.24-7.62	0.002
HbA1c ≤7.0% (53.0 mmol/mol)without hypoglycaemia	5.48	1.71-17.51	0.0003	2.68	0.82-8.71	0.04	3.69	1.36-9.96	0.001	5.48	2.01-14.88	<0.0001
HbA1c ≤7.0% (53.0 mmol/mol) without weight gain	5.39	1.83-15.88	0.0004	1.57	0.5-4.85	0.01	2.14	0.86-5.3	<0.0001	3.08	1.24-7.62	<0.0001
HbA1c ≤7.0% (53.0 mmol/mol) without hypoglycaemia or weight gain	10.93	1.51-78.95	0.003	5.43	1.15-25.62	0.007	9.66	2.63-35.43	<0.0001	10.44	2.64-41.17	<0.0001

Models restricted to baseline metformin users and adjusted for sex, age, baseline HbA1c, diabetes duration, body weight, and body mass index using generalized least squares regression (n = 273 people). OR [95% CI] presented for IDegLira/ICT.

CI, confidence interval; ICT, intensified conventional insulin treatment; IDegLira, insulin degludec/liraglutide; OR, odds ratio
